# Impact of New Standardized Population for Estimating Cancer Incidence in Indian Context- an Analysis from National Cancer Registry Programme (NCRP) 

**DOI:** 10.31557/APJCP.2020.21.2.371

**Published:** 2020

**Authors:** Sathishkumar K, Vaitheeswaran K, Stephen S, Sathya N, Prashant Mathur

**Affiliations:** *National Centre for Disease Informatics and Research, Indian Council of Medical Research (ICMR), Bangalore, India. *

**Keywords:** Indian standard population, age adjusted rate, age standardised Rrate, NCRP, national population

## Abstract

**Objective:**

Standardization adjusts for variations in population age-distribution and provides a summary measure for the comparison of populations and comparisons of time-trends in population. Globally, several standard population were used by many countries for comparison of rates. Segi World Standard Population (WSP) is suitable for international comparison. However, national standard population would be more appropriate for Intra-national comparison as the standard should be similar to the population of interest. This study aimed to develop Indian Standard Population (ISP) for estimating Age Adjusted Rate (AAR) and, to understand the outcome on the cancer incidence rate using ISP in Population Based Cancer Registries (PBCRs).

**Methods:**

The Indian Standard Population was estimated based on the average age structure of three Censuses (1991,2001 and 2011). Age Adjusted Rate has been estimated using two standard populations of WSP and ISP for magnitude of change and comparison between 15 PBCRs in India.

**Results:**

The implementation of ISP led to overall 20% reduction in AAR for all sites of cancers in both sexes and minor relative changes in the ranking among PBCRs. Time trends in cancer incidence rate showed same temporal pattern curve using both ISP and WSP, AARs.

**Conclusion:**

The ISP would be more representative of the age-structure of Indian registries population and this would give more realistic comparison across the different PBCRs in India.

## Introduction

Comparing incidence and mortality rates in two or more different regions/areas is vital for the assessment of health status of the community (Naing, 2000). A comparison between crude rates would be less informative as they are not very informative about the health status of a population especially for cancer which is more dependent on age. Age is the highest risk for developing cancer and the risk of epithelial cancers, which comprise 90% of all cancers worldwide, increases approximately as a fifth power of age (Armitage and Doll, 1954). Age is the most common variable used for standardization. Globally, standardization is the procedure used to adjust for differences in population structure and provides a single summary measure for the comparison of different populations/areas. Generally, a National population is considered as “Standard Population” for comparing rates in different geographic regions/populations within the country.

There are two commonly used methods for calculating standardized rates that is direct and indirect standardization. A common age-structured population is used as standard population in direct standardization. This could be the existing population of the country (e.g., United States population, 1999, Australian Standard Population, 2001) or may be arbitrary. A common set of age specific rate is applied to the population’s age structure whose rates are to be standardized which is known as indirect standardization. The most common and simple technique is the direct age standardization method (Mausner and Bahn, 1974).

The world standard population was previously used by many studies for comparing cancer incidence rates in different regions (NCDIR, 2013; Torre et al., 2015).This was originally proposed in 1960 as the pooled population of 46 countries and, thereafter, revised in 1966.The new standard population in the year 2000 was constructed by World Health Organisation (WHO) as the average of projected world population age structure during the years 2000–2025 (Segi, 1960; Doll et al., 1966; Ahmad et al., 2000).The US has replaced the existing 1940 standard population with new standard of US 2000 population (Anderson and Rosenberg, 1998). Some other standard populations which have extensively been used are those of Canada; Nordic countries (NORDCAN population in 2014); Australia (Australian Standard Population, 2001); the International Network for the Demographic Evaluation of Populations and Their Health (INDEPTH) standard population for low and middle-income countries and the European standard population for all cause of death and cancer incidence (Waterhouse 1976; Government of Canada, 1991; Engholm et al., 2016; HealthStats NSW, 2017; Sankoh et al., 2014; Crocetti et al., 2016).

The National Centre for Disease Informatics and Research (NCDIR), National Cancer Registry Programme (NCRP) in India was initiated by the Indian Council of Medical Research (ICMR) with a network of cancer registries across the country in December 1981 and it has expanded to 36 Population Based Cancer Registries (PBCRs). NCDIR-NCRP reports Age Adjusted Cancer incidence rates based on Segi World Standard Population (NCDIR, 2013, 2016).

Since, Segi standard population (Segi, 1960) age structure is not representative of Indian population structure, an attempt was made in this study to use Indian Standard Population (ISP) for estimating Age Standardised Rate (ASR)/Age Adjusted Rate (AAR) using age structure of Indian population and, to understand the outcome on the incidence rate of cancer using Indian standard population in Population Based Cancer Registries along with Segi WSP (Segi, 1960).

## Materials and Methods

Census of India is the single largest source of a variety of statistical information on various characteristics of the people of India. The source data for Indian standard population was derived from Census of India (Census of India, 2018). The ISP was conceptualized based on average age structure of Indian population with the age group of (0-4,5-9,10-14,…75+) of population of last three censuses (1991, 2001 and 2011).The last three census population by age group for both sexes were added and relative proportion were arrived at. Since cancer incidence rate multiplication factor is 100,000, the proportion of age groups are multiplied by 100,000 and rounded with nearest five hundreds. A crude incidence rate is the number of new cancers occurring in a specified population in a specified period, usually expressed per 100,000 population at risk. Age Specific Incidence Rate was the number of new cancers occurring in a given age group (eg. 10-14) in specified population and period. Age specific rate was multiplied by the standard population of that same age group. The age-specific results are added up to get the age-adjusted incidence rate for the area of study. This is called direct method of standardization (Naing, 2000).

The NCRP population Based Cancer Registries in India, collect all new cancers occurring in the resident population of defined geographic area through active case-finding procedures and principles advocated by the International Agency for Research on Cancer, the International Association of Cancer Registries (Jensen and Storm, 1991) based on multiple sources of data such as Government Hospitals, Private Hospitals, Nursing Homes, Clinics, Diagnostic Labs, Imaging centres, Hospices and Registrars of Births and Deaths (NCDIR, NCRP, 2018).

We have used the “Three-year report of Population Based Cancer Registries (PBCR):2012-2014 data as the source to calculate Crude rate (CR), Age adjusted rate using WSP and ISP for all sites, stomach, breast, cervix, prostate and myeloid leukaemia as varied risk patterns across the age group (NCDIR, 2016). Based on the AAR of two standard populations, the ranking of the cancer registries were compared. We estimated the Spearman rank correlation (r) between the rates using WSP and ISP. Also, the percentage change between AAR of world standard population and ISP has been observed by cancer site wise.

## Results

**Figure 1:**
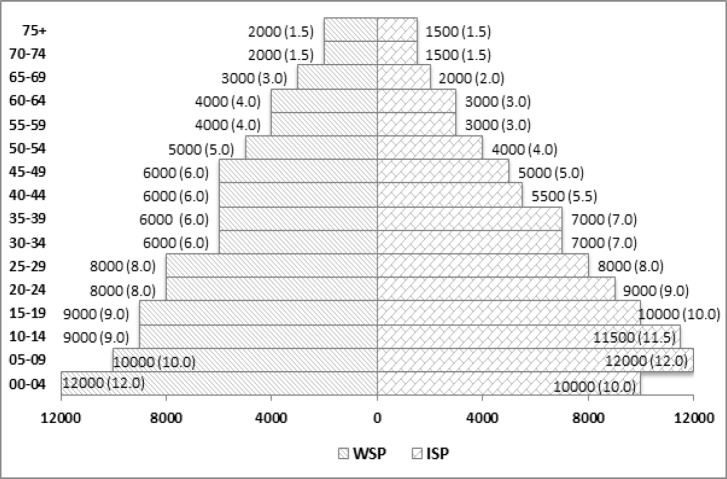
Distribution of World Standard population (WSP) and Indian Standard Population (ISP) per 100,000 population (%)

Figure 1.ISP had higher population in younger age group compared with WSP except in (0-4) age group. Above 45 year age the lower population (20%) were seen in ISP compared with WSP (26%). 

Table 1 provides Crude rate, Age Adjusted Rate using WSP and ISP for the first 15 PBCRs (based on AAR-WSP ranking) for all sites of cancer (ICD-10: C00-C97) by sex. The first 15 PBCRs were ranked based on AAR-WSP and AAR-ISP rate as well. There was only one position change in ranking otherwise we did not find major changes in relative ranking of the PBCRs using either WSP or ISP. However, there was a change in magnitude of standardized rate using ISP, for example the incidence rate Aizawl district of Mizoram State (North eastern state of India) using WSP 270.7 per 100,000 reduced to 210.2 per 100,000 using ISP in males. The Papumpare district reduced from ASR 249.0 per 100,000 to 200.5 per 100,000 using ISP in females. As an average there was 20% of reduction in AAR across all the registries.

Table 2 provides the magnitude of change in ASR using two different population for stomach cancer (ICD-10: C16) by gender. There was no change in relative ranking for stomach cancers in PBCRs using both the standard population. 

Table 3. The leading site of breast cancer (ICD-10: C50) in females showed major changes in the ranking using ISP- standardized rate. The first five ranking based on WSP (Delhi, Chennai, Bangalore, Thiruvananthapuram and Mumbai) were changed to (Delhi, Chennai, Thiruvananthapuram, Patiala, and Bangalore). However, the difference in rates between PBCRs are very minimum. The second leading sites of cervical cancer in (ICD-10: C53) females showed relative change in rank at 11th position. 

Table 4. Prostate cancer (ICD-10: C61) does not show any change in the ranking using either standard population. Though myeloid leukaemia (ICD-10: C92-C94) in males showed changes in the ranking, the difference in rates are minor. The Spearman rank correlation (r) between the rates using WSP and ISP showed positive correlation in all sites and specific sites of cancer.

Table 5. In percentage distribution by age group of different registries population as per Census 2011, the PBCRs of Chennai, Kollam, Mumbai, Nagpur and Patiala had higher older age (65+) population than ISP which resulted CR becomes greater than ISP-AAR and Meghalaya had lower older age population than ISP which resulted CR becomes lesser than ISP-AAR. Mizoram, Bhopal and Delhi percentage of older age population is closer to ISP which resulted CR and ISP-AAR becomes closer. 

**Figure 2:**
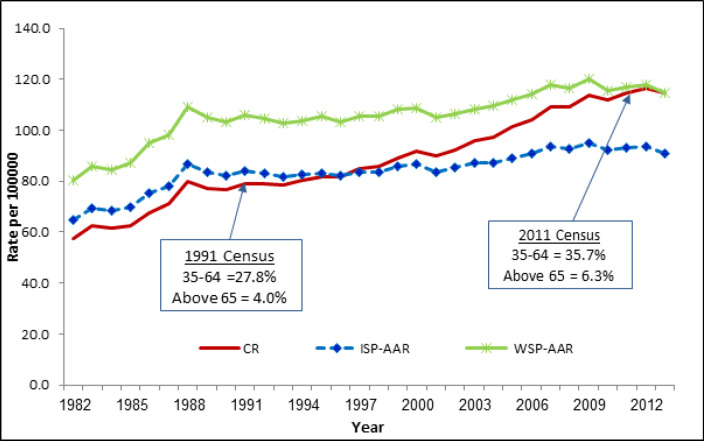
Trends in Crude Rate (CR), Age Adjusted Rate (AAR) using World Standard Population (WSP) and Indian Standard Population (ISP) for all sites of Cancer (Males) - Chennai (1982-2013)

Figure 2. Trends in Age Adjusted Rate using ISP and WSP for All sites of Cancer showed same temporal pattern curve and the ISP-AAR incidence rate is lesser than WSP-AAR over the period (1982-2013). At the beginning period the CR was lower than ISP-AAR and it crossed over to higher side in the year 1997. The percentage of older age group (65+) population in Chennai PBCR increased to 6.3% in 2011 from 4.0% in 1991. 

**Table 1:** Crude Rate (CR) and Age Adjusted Rate (AAR) Per 100,000 Population Using World Standard Population (WSP) and Indian Standard Population (ISP) in Population Based Cancer Registries (PBCR) – Males and Females (All Sites)

	Male							Female					
Registry	CR	Rank*	AAR-WSP	AAR-ISP	Rank*	ISP-WSP (AAR) (%)	Registry	CR	Rank*	AAR-WSP	AAR-ISP	Rank	ISP-WSP (AAR) (%)
Aizawl	204.6	1	270.7	210.2	1	-22.3	Papumpare	115.3	1	249.0	200.5	1	-19.5
Papumpare	103.5	2	230.4	181.1	2	-21.4	Aizawl	167.3	2	207.7	165.6	2	-20.3
East K.Hills	125.5	3	218.3	173.1	3	-20.7	Kamrup Urban	123.3	3	174.0	137.9	3	-20.7
Mizoram State	147.4	4	211.5	163.5	4	-22.7	Mizoram State	121.7	4	165.8	132.7	4	-20.0
Kamrup Urban	143.4	5	206.0	158.3	5	-23.2	Delhi	121.7	5	144.8	118.2	5	-18.4
Meghalaya	88.8	6	169.6	134.5	6	-20.7	Chennai	132.3	6	126.2	101.7	6	-19.4
Delhi	112.6	7	149.4	119.3	7	-20.1	Bangalore	106.5	7	125.9	100.4	7	-20.3
Thi'puram	160.7	8	132.0	104.2	8	-21.1	Thi'puram	153.9	8	120.4	99.6	8	-17.3
**Nagaland**	74.7	9	**125.8**	**98.6**	**10**	**-21.6**	Mumbai	117.0	9	118.5	94.6	9	-20.2
**Cachar**	96.2	10	**125.4**	**98.9**	**9**	**-21.1**	East K.Hills	75.0	10	117.0	92.4	10	-21.0
Kollam	148.0	11	120.5	94.7	11	-21.4	Patiala	112.8	11	111.2	91.1	11	-18.1
Chennai	115.5	12	116.1	92.3	12	-20.5	Bhopal	90.4	12	108.3	87.8	12	-18.9
Mumbai	98.1	13	113.1	88.8	13	-21.5	Imphal West	100.3	13	103.6	85.4	13	-17.6
Pasighat	83.1	14	107.4	86.7	14	-19.3	**Kolkata**	120.8	14	**103.4**	**84.0**	**15**	**-18.8**
Bangalore	82.8	15	105.4	82.1	15	-22.1	**Kollam **	130.3	15	**101.7**	**84.5**	**14**	**-16.9**
Correlation (r)	0.99						Correlation (r)	0.99					

East K.Hills - East KhasiHills;Th'puram- Thiruvananthapuram; Change in rank are highlighted in bold; *Rank- Ranking status of PBCR based on WSP and ISP; ISP-WSP (AAR) (%) – Change in percentage between ISP and WSP AAR’s

**Table 2:** Cancer Incidence: Crude Rate (CR) and Age Adjusted Rate (AAR) Per 100,000 Population Using World Standard Population (WSP) and Indian Standard Population (ISP) in Population Based Cancer Registries (PBCR) –Stomach (ICD-10:C16) - Males and Females

Stomach-Males							Stomach-Females						
Registry	CR	Rank*	AAR- WSP	AAR- ISP	Rank*	ISP-WSP (AAR) (%)	Registry	CR	Rank*	AAR- WSP	AAR-ISP	Rank*	ISP-WSP (AAR) (%)
Papumpare	21.5	1	50.2	39.0	1	-22.3	Papumpare	11.4	1	29.2	22.9	1	-21.6
Aizawl	31.8	2	43.9	33.3	2	-24.1	Aizawl	17.4	2	23.7	18.0	2	-24.1
Mizoram State	27.3	3	41.1	31.1	3	-24.3	Mizoram State	13.7	3	20.1	15.5	3	-23.3
Naharlagun	14.3	4	26.7	20.9	4	-21.7	Naharlagun	8.1	4	16.3	12.7	4	-22.1
Pasighat	16.2	5	22.8	17.3	5	-24.1	Pasighat	8.4	5	12.2	9.8	5	-19.7
Nagaland	9.2	6	17.8	13.5	6	-24.2	Nagaland	5.9	6	11.1	8.7	6	-21.6
Sikkim State	11.3	7	14.8	11.2	7	-24.3	Kamrup Urban	6.1	7	8.6	6.8	7	-21.8
East K Hills	7.3	8	14.3	11.0	8	-23.1	East K Hills	5.1	8	7.6	6.1	8	-19.7
Kamrup Urban	9.6	9	14.1	10.6	9	-24.8	Meghalaya	4.0	9	6.8	5.5	9	-19.1
Meghalaya	6.1	10	12.5	9.7	10	-22.4	Sikkim State	4.5	10	6.8	5.1	10	-25.0
Chennai	10.8	11	10.8	8.3	11	-23.1	Chennai	5.4	11	5.1	4.0	11	-21.6
Bangalore	6.3	12	8.2	6.2	12	-24.4	Bangalore	3.8	12	4.7	3.6	12	-23.4
Dibrugarh	5.7	13	7.6	5.9	13	-22.4	Dibrugarh	3.6	13	4.3	3.6	13	-16.3
Kollam	6.9	14	5.5	4.2	14	-23.6	Imphal West	2.6	14	2.8	2.2	14	-21.4
Cachar	4.0	15	5.4	4.2	15	-22.2	Cachar	2.0	15	2.6	2.1	15	-19.8
Correlation (r)	1.00						Correlation (r)	1.00					

East K.Hills - East Khasi Hills;*Rank- Ranking status of PBCR based on WSP and ISP; ISP-WSP (AAR) (%) – Change in percentage between ISP and WSP AAR’s

**Table 3:** Crude Rate (CR) and Age Adjusted Rate (AAR) Per 100,000 Population Using World Standard Population (WSP) and Indian Standard Population (ISP) in Population Based Cancer Registries (PBCR) – Breast(ICD-10:C50) and Cervix (ICD-10:C53) - Females

	Breast (ICD-10:C50)						Cervix (ICD-10:C53)						
Registry	CR	Rank*	AAR-WSP	AAR- ISP	Rank*	ISP-WSP (AAR) (%)	Registry	CR	Rank*	AAR-WSP	AAR- ISP	Rank*	ISP-WSP (AAR) (%)
Delhi	34.8	1	41.0	33.2	1	-19.0	Papumpare	15.6	1	30.2	24.9	1	-17.5
Chennai	40.8	2	37.9	30.6	2	-19.3	Aizawl	26.0	2	28.0	24.4	2	-12.9
**Bangalore**	29.3	3	**34.4**	**27.4**	**5**	**-20.3**	Mizoram	19.4	3	23.1	20.1	3	-13.0
**Th'puram**	43.9	4	**33.7**	**27.6**	**3**	**-18.1**	Pasighat	18.7	4	22.5	19.6	4	-12.9
**Mumbai**	33.6	5	**33.6**	**26.8**	**7**	**-20.2**	Barshi rural	17.7		16.1	12.7	5	-21.1
**Patiala**	34.2	6	**33.1**	**27.5**	**4**	**-16.9**	Chennai	16.7	6	15.9	12.6	6	-20.8
**Bhopal**	28.2	7	**33.0**	**27.1**	**6**	**-17.9**	Delhi	13.2	7	15.5	12.5	7	-19.4
**Nagpur**	30.4	8	**29.3**	**24.5**	**9**	**-16.4**	Bangalore	13.1	8	15.3	12.2	8	-20.3
**Papumpare**	17.3	9	**29.2**	**25.0**	**8**	**-14.4**	Barshi Exp	15.6	9	14.7	11.9	9	-19.0
Aizawl	24.3	10	28.0	23.5	10	-16.1	**Kamrup Urban**	10.6	10	**14.5**	**11.5**	**11**	**-20.7**
Kollam	36.3	11	27.7	23.0	11	-17.0	**Aurangabad**	12.0	11	**14.3**	**11.7**	**10**	**-18.2**
Kamrup	21.5	12	27.1	22.1	12	-18.5	Bhopal	11.3	12	13.8	11.1	12	-19.6
Pune	23.4	13	26.4	21.0	13	-20.5	Nagaland	8.9	13	13.1	11.0	13	-16.0
Kolkata	30.7	14	25.5	21.0	14	-17.6	**Nagpur**	12.9	14	**12.9**	**10.4**	**15**	**-19.4**
Ahmedabad U	23.0	15	23.8	19.3	15	-18.9	**Cachar**	10.9	15	**12.7**	**10.9**	**14**	**-14.2**
Correlation (r)	0.97						Correlation (r)	0.99					

Th'puram- Thiruvananthapuram; Ahmedabad U- Ahmedabad Urban; Barshi Exp- Barshi Expanded; Change in rank are highlighted in bold; *Rank- Ranking status of PBCR based on WSP and ISP; ISP-WSP (AAR)(%) – Change in percentage between ISP and WSP AAR’s

**Table 4:** Cancer Incidence: Crude Rate (CR) and Age Adjusted Rate (AAR) Per 100,000 Population Using World Standard Population (WSP) and Indian Standard Population (ISP) in Population Based Cancer Registries (PBCR) – Prostate (ICD-10:C61) and Myeloid Leukaemia (ICD-10:C92-C94) – Males

Prostate							Myeloid Leukaemia						
Registry	CR	Rank*	AAR-WSP	AAR- ISP	Rank*	ISP-WSP (AAR) (%)	Registry	CR	Rank*	AAR-WSP	AAR- ISP	Rank*	ISP-WSP (AAR) (%)
Delhi	7.6	1	12.4	9.1	1	-26.6	Delhi	2.8	1	3.2	2.9	1	-9.4
Kamrup	6.9	2	12.2	8.9	2	-27.0	**Patiala **	2.9	2	**2.9**	**2.6**	**3**	**-10.3**
Mumbai	7.7	3	9.8	7.2	3	-26.5	**Imphal West**	2.8	3	**2.8**	**2.7**	**2**	**-3.6**
Thi'puram	11.5	4	9.4	6.9	4	-26.6	**Thi'puram**	3.1	4	**2.7**	**2.5**	**5**	**-7.4**
Kolkata	9.8	5	8.2	6.0	5	-26.8	**Kollam**	3.0	5	**2.7**	**2.5**	**4**	**-7.4**
Bangalore	5.8	6	8.2	6.0	6	-26.8	**Aizawl**	2.2	6	**2.7**	**2.2**	**7**	**-18.5**
Chennai	6.2	7	6.7	4.9	7	-26.9	**Bangalore**	2.3	7	**2.6**	**2.2**	**6**	**-15.4**
Papumpare	1.4	8	6.6	4.9	8	-25.8	**Ahmedabad U**	2.4	8	**2.4**	**2.2**	**8**	**-8.3**
Patiala	5.8	9	6.3	4.7	9	-25.4	**Chennai **	2.4	9	**2.3**	**2.0**	**10**	**-13.0**
Pune	4.5	10	6.3	4.6	10	-27.0	**Bhopal **	2.2	10	**2.2**	**2.1**	**9**	**-4.5**
Kollam	7.6	11	6.1	4.5	11	-26.2	**Kolkata**	2.4	11	**2.1**	**2.0**	**11**	**-4.8**
Bhopal	4.0	12	5.6	4.1	12	-26.8	**Sikkim State **	1.9	12	**2.0**	**1.8**	**13**	**-10.0**
Ahmedabad U	3.3	13	4.4	3.2	13	-27.3	**Wardha**	2.1	13	**1.9**	**1.9**	**12**	**0.0**
Aizawl	2.6	14	3.5	2.6	14	-25.7	**Mumbai**	1.7	14	**1.7**	**1.5**	**15**	**-11.8**
Mizoram State (MZ)	2.0	15	3.0	2.2	15	-26.7	**Barshi Rural **	1.6	15	**1.7**	**1.6**	**14**	**-5.9**
Correlation (r)	0.99						Correlation (r)	0.97					

Th'puram- Thiruvananthapuram; Ahmedabad U- Ahmedabad Urban; Change in rank are highlighted in bold; *Rank- Ranking status of PBCR based on WSP and ISP; ISP-WSP (AAR) (%) – Change in percentage between ISP and WSP AAR’s

**Table 5:** Percentage Distribution by Age Group of Population (Census 2011) with Cancer Incidence Rate (All sites of cancer - Both sexes) in Selected Population Based Cancer Registries and Indian Standard Population (ISP)

Registry / Zone	Age Group				CR	AAR-WSP	AAR-ISP
	0-14	15-34	35-64	65+			
**South**							
** ISP**	**34.2**	**34.3**	**26.6**	**4.9**	-	-	-
Chennai	21.5	36.5	35.7	6.3	123.9	120.6	96.6
Kollam	22.1	30.0	39.0	8.8	138.5	110.1	89.1
**Central**							
Bhopal	29.1	38.4	27.8	4.8	86.2	104.5	84.3
**West**							
Mumbai	22.1	39.2	33.2	5.5	106.7	115.1	91.0
Nagpur	23.4	37.1	33.6	5.8	90.2	91.6	75.6
**North**							
Delhi	27.1	39.0	29.9	4.0	116.4	146.4	118.2
Patiala	25.4	37.5	30.8	6.4	101.1	104.3	84.5
**North East**							
Meghalaya	40.5	35.7	20.8	3.0	71.5	130.4	103.7
Mizoram	32.5	37.6	25.9	4.0	134.6	188.8	148.2

CR, Crude Rate; WSP, World Standard Population; ISP, Indian Standard Population

## Discussion

The main reason for using Indian standard population is that it is more representative of Indian registries population and the most recent available population data. The WSP give more weightage to the older population (most of the cancers occur) than ISP. The larger the difference between the age distributions of the standard population and the study population, the greater the difference in the crude and adjusted rates for the study population. Though WSP available to compare the rate with other population, we also need to have standard population which is of close to the real population. Therefore, many countries have used national standard population for estimating AAR. ISP will help in making sub-national comparison.

The results of this preliminary analysis using the Indian Standard Population on selected sites of cancer rates showed significant differences as compared with the Segi World population. There was a major difference in the age structure of WSP compared to ISP, especially in the older age group. In general, the use of a young standard (higher proportion of young population) leads to a low standardised mortality rate, and an old standard to a high overall rate, due to the strong positive association between age and mortality (Robson et al., 2007).

Standardization may be used to adjust for confounding variables in which age and gender are two of the most common variables. The standard population based on gender was not done because the differences in age-structure were not substantial. The standard population can affect the magnitude of the adjusted rate, the observed trends in adjusted rates over time and ranking of priorities (e.g., ranking of leading site of cancer). Since the standard population has a major impact on the results and the conclusions drawn, one should select the standard population very carefully before analyzing rates (Kitagawa, 1964; Choi et al., 1999).

NCDIR-NCRP registries were using Segi world standard population since from its inception in 1981 (Segi, 1960; Doll et al., 1966). WHO adopted a standard based on the average world population age-structure for the period 2000-2025.The WHO standard has fewer children proportionally and a greater proportion of adults aged 70 years compared to the Segi world standard (Ahmad et al., 2000). The US-2000 and Canada-1992 standard population was projected based on the census (Anderson and Rosenberg, 1988; Government of Canada, 1991; Anderson and Rosenberg, 1998). European-1976 standard population was based on several Scandinavian population and the revision of European population was published in 2016 based on the 2011–2030 population projections of the unweighted average age structure of the populations of EU-27 member states plus the European Free Trade Association (Waterhouse et al., 1976; European Commission, 2013). Similarly, the ISP is based on average age structure of the India populations of last three censuses (1991, 2001 and 2011). The census data was considered to be a true estimate rather than using hypothetical projected population. Before comparing various different populations, a ‘pooled’ standard population (which is created by adding together the populations of the areas/period being compared) would reduce the variance of the standardized rates (Rosenberg et al., 1992; Choi et al., 1999).

The National Cancer Registry Programme has been in existence from 1982 with three PBCRs and today its coverage has expanded to 36 PBCRs with about 10% of the population in India being covered. Since the population coverage by cancer registries were expanding periodically, the total India census population as considered as standard rather than pooled PBCRs population. Many PBCRs (Aizawl, Bangalore, Bhopal, Cahcar, Delhi and Pasighat) CR close to ISP-AAR indicates that, especially the older age population structure of PBCRs similar to the ISP.

Breast cancer is the leading site of cancer among women in India (NCDIR, 2016). The changing of rank was seen by changing the standard population in specific sites of cancer including breast. Population Based Cancer Registries can measure cancer incidence, its trend and mortality and have a unique role in planning and evaluating cancer control plans and reducing the cancer burden in the community (Bray et al., 2015). Based on the change in rank, planning cancer prevention, cancer control activities and allocation of resources for health interventions between the regions / states can be re-prioritised in India. A better local policy decision and rolling out programme can be done at our own population.

GLOBOCAN has not replaced WSP with WHO due to various reasons (Bray et al., 2002). This study aimed to compare the WSP with ISP. Our result suggest that for comparison of AAR between Indian registries the ISP may be used and, for comparison of AAR between countries the WSP population may be used. The need for implementing ISP was due to the major difference in AAR between ISP and WSP, relative changes in the ranking and this is the national standard. 

The time trends in cancer incidence rate with the effect of ISP and WSP on all sites of cancer for the period 1982-2013 showed that parallel curves as seen in other studies. A time trend in CR curve was lower than the ISP-AAR at the beginning period of 1982-1996 and later the CR over took ISP-AAR. This change in curve was due to gradual increase in the older age population in the registry area.

Norwegian registry has done major change in the 2014 report as preferred a Norwegian Standard Population, instead of the World Standard Population to estimate age-standardised rates. The higher weights in the oldest age groups of Norway 2014 reference population led to twice as high age-standardised rates (Cancer Registry of Norway, 2015). The use of Revised European Standard Population, shown slight impact on the pattern of time trends and the relative ranking of countries compared to European Standard Population (Crocetti et al., 2016).

The artificial WSP population was defined in the 1960s, where 62% of the population was assumed to be below 35 years of age, and 38% above age 35. This age structure is different from India (as per Census, 2011), where a higher percentage of the population was below 35 (69%) and less population in above 35 (31%) years of age (Census of India, 2018).

One should change the future standard population if the differences between the standard and actual population become problematic (Anderson and Rosenberg, 1998). The revision of standard population would be based on the future release of Census of India, population, subject to major significant changes in the age structures. Statistical adjustment was performed in epidemiology to remove or reduce the confounding effects of extraneous confounding factor, for example age, when comparing Incidence or mortality rates in different populations. An actual measure of summary information of Age Adjusted rate using ISP may be better in health planning and policy for comparison of different population and time period. Since India sub-population are remarkably different from the Segi standard the simultaneous observation of crude rate, age adjusted rate using world standard and national standard would offer better information.

In conclusion, Indian Standard population would be more representative of the age structure for many Indian registries and this would give more realistic comparison between populations within India. This information will be useful for cancer surveillance, policy and program development. Achieving entire national coverage of cancer registration in India would result more closer crude rate with ISP-AAR.
